# Origin of Solvent Dependency of the Potential of Zero Charge

**DOI:** 10.1021/jacsau.3c00552

**Published:** 2023-11-15

**Authors:** Weiqiang Tang, Shuangliang Zhao, Jun Huang

**Affiliations:** † State Key Laboratory of Chemical Engineering and School of Chemical Engineering, East China University of Science and Technology, Shanghai 200237, China; ‡ Institute of Energy and Climate Research, IEK-13: Theory and Computation of Energy Materials, Forschungszentrum Jülich GmbH, Jülich 52425, Germany; § Guangxi Key Laboratory of Petrochemical Resource Processing and Process Intensification Technology and School of Chemistry and Chemical Engineering, Guangxi University, Nanning 530004, China; ∥ Theory of Electrocatalytic Interfaces, Faculty of Georesources and Materials Engineering, RWTH Aachen University, Aachen 52062, Germany

**Keywords:** solvent effect, electron spillover, density-potential functional theory, potential of zero charge, differential double layer capacitance

## Abstract

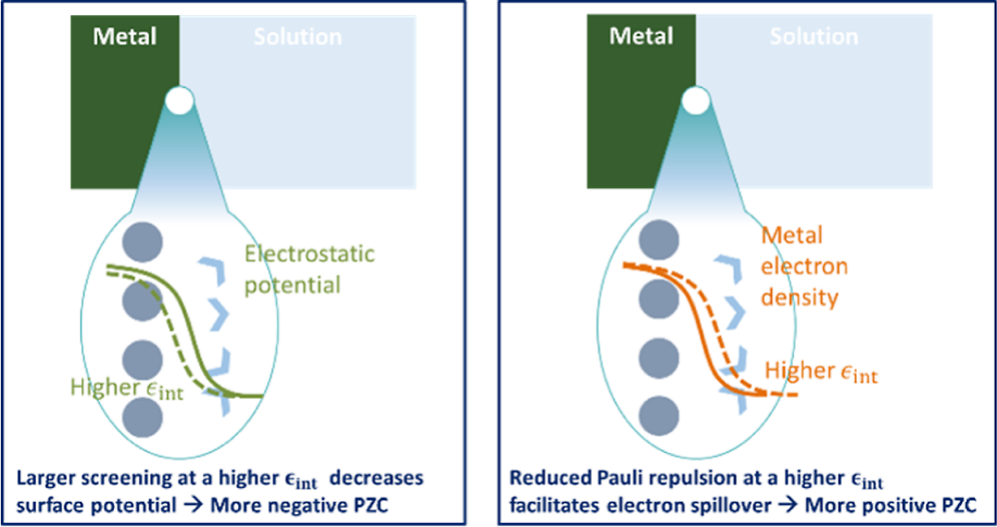

Fundamental properties of the Au(111)–KPF_6_ interface, particularly the potential of zero charge (PZC), exhibit pronounced variations among solvents, yet the origin remains largely elusive. In this study, we aim to link the solvent dependency to the microscopic phenomenon of electron spillover occurring at the metal–solution interface in heterogeneous dielectric media. Addressing the challenge of describing the solvent-modulated electron spillover under constant potential conditions, we adopt a semiclassical functional approach and parametrize it with first-principles calculations and experimental data. We unveil that the key variable governing this phenomenon is the local permittivity within the region approximately 2.5 Å above the metal edge. A higher local permittivity facilitates the electron spillover that tends to increase the PZC on the one hand and enhances the screening of the electronic charge that tends to decrease the PZC on the other. These dual effect lead to a nonmonotonic relationship between the PZC and the local permittivity. Moreover, our findings reveal that the electron spillover induces a capacitance peak at electrode potentials that are more negative than the PZC in concentrated solutions. This observation contrasts classical models predicting the peak to occur precisely at the PZC. To elucidate the contribution of electron spillover to the total capacitance, we decompose the total capacitance into a quantum capacitance of the metal *C*_q_, a classical capacitance of electrolyte solution *C*_c_, and a capacitance *C*_qc_ accounting for electron–ion correlations. Our calculations reveal that *C*_qc_ is negative due to the promoted electron spillover at more negative potentials. Our work not only reveals the importance of local permittivity in tuning the electron spillover but also presents a viable theoretical approach to study solvent effects on electrochemical interfaces under operating conditions.

## Introduction

In its infancy, Kohn–Sham density functional theory (DFT) had been employed to describe the charge density, work function, and surface energy of metal surfaces. These works revealed the crucial importance of electron spillover (or spillout in plasmonics literature^1,2^), namely, flow of electrons over the edge of metal surfaces, in determining these properties of metal surfaces. For example, the work function Φ is calculated as, Φ = *e*_0_ χ_m_ – μ_e_, where χ_m_ is the electrostatic potential difference from metal bulk (*x* = −∞) to far vacuum (*x* = ∞) and μ_e_ is the chemical potential of electrons in metal bulk. While the latter is a bulk property independent of the electron distribution near the metal surface, the former is calculated as,  with the vacuum permittivity *ϵ*_0_, electron density *n*(*x*), and charge background density *n*_+_(*x*).^3,4^ It is readily seen that by tuning *n*(*x*) one can change χ_m_ and thereby Φ. It has been recently recognized that the same electron spillover also influences the surface-plasmon resonance of nanoparticles.^5−7^

When a metal is brought into contact with an electrolyte solution, the electron spillover is expected to be different from the preceding scenario of a metal–vacuum interface.^8^ The changes are caused by direct interactions, such as Pauli repulsion between metal electrons and solvent electrons on the one hand, and indirect interactions, such as the screened electrostatic field due to solvent polarization on the other.^9,10^ Using *ab initio* molecular dynamics (AIMD) simulations, one can “visualize” such changes at the atomistic scale.^11,12^ For example, recent AIMD simulations^10,11,13^ reveal that electron accumulation on the metal side and electron depletion on the water side are responsible for the lower potential of zero charge (PZC) at the metal–water interface than at the metal–vacuum interface. Specifically, interfacial water molecules with the O-down configuration in the first layer push the spilled electrons back into the metal skeleton through Pauli repulsion.^14,15^ Such changes are termed solvent-modulated electron spillover at the metal–solution interface. In addition to the PZC, the solvent-modulated electron spillover also determines the double-layer capacitance (*C*_dl_).^8,16,17^ A relevant concept describing the effect of electron spillover on *C*_dl_ is the quantum capacitance or electronic capacitance.^18−21^

Limited by the computational cost, AIMD studies are usually conducted at the uncharged metal–solution interface with a “simple” solvent, viz., water.^22−25^ Solvent-modulated electron spillover in other types of solvents, such as aprotic solvents, is much less understood. One can proceed a step further by adding a few nonspecifically adsorbing cations or anions into the system, so as to simulate charged metal–solution interfaces.^25−27^ Due to the relatively small simulation size that can be afforded practically, the fluctuation in the electrode potential could be up to 0.5 V, and addition of one cation or anion into a 6 × 6 Pt(111) surface supercell is tantamount to a change of the electrode potential of more than 0.1 V.^28,29^ Therefore, a computationally efficient constant-potential approach is certainly welcome to understand the solvent-modulated electron spillover phenomena under constant potential conditions in a wider range of solvents. This work is an attempt in this direction.

**Figure 1:**
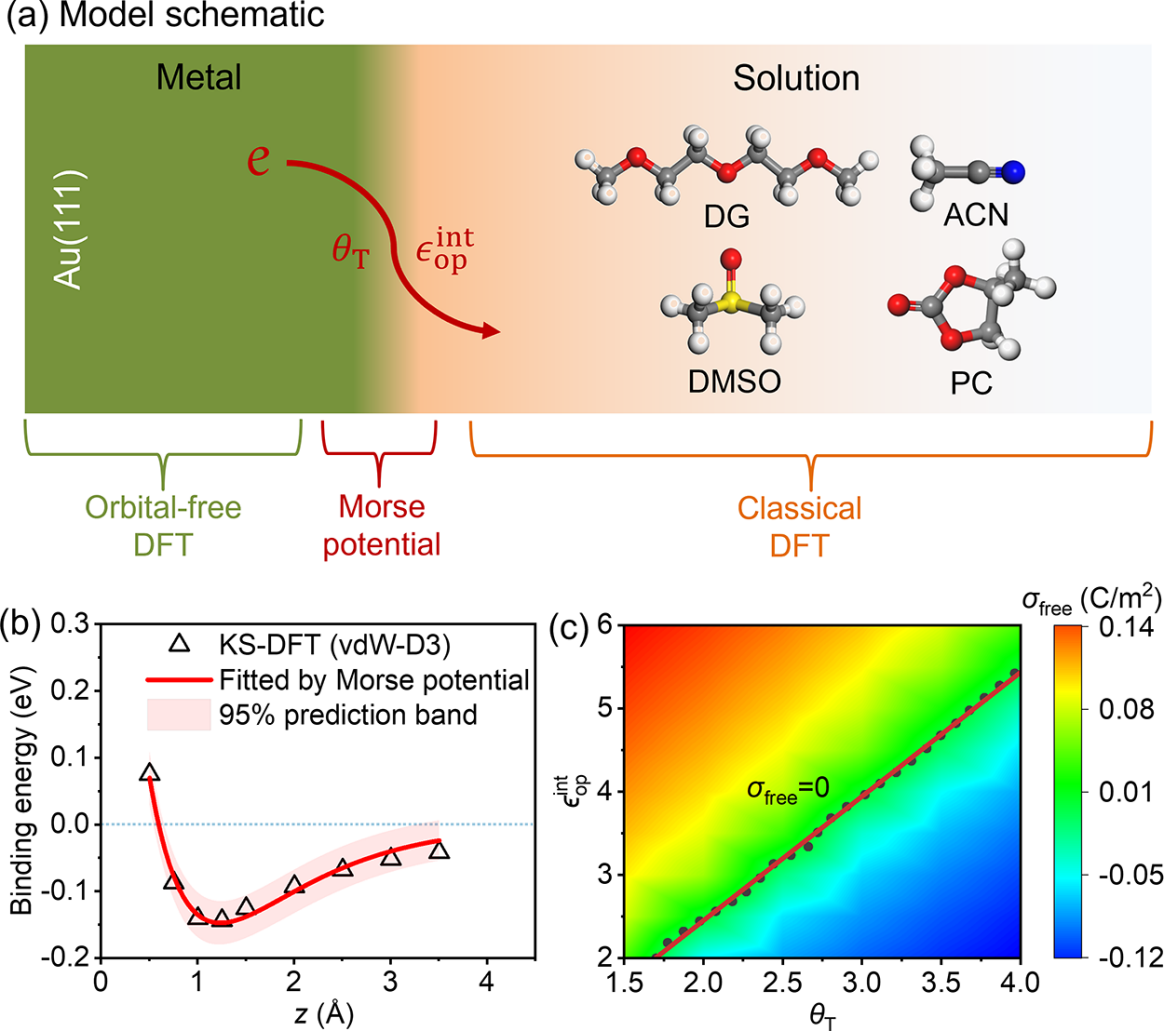
Model schematic and parametrization. (a) Schematic of the Au(111)–solution interface described by DPFT. The metal side, solution side, and short-range interactions between the metal and solution are described by the orbital-free DFT, classical DFT, and Morse potential, respectively. The electron spillover is influenced by the gradient coefficient in the kinetic energy functional *θ*_T_ and interface optical permittivity *ϵ*_op_^int^. In accordance with an experimental study,^33^ four aprotic solvents, DG, ACN, DMSO, and PC, are examined. The white, gray, red, blue, and yellow balls represent H, C, O, N, and S atoms, respectively. (b) Parameterization of the Morse potential between Au(111) and ACN molecules. The empty triangles represent the binding energy calculated from Kohn–Sham DFT, while the solid red lines represent the fitting Morse potential. Morse potentials for other electrolyte species are provided in Table S2. (c) Determination of the *θ*_T_ and *ϵ*_op_^int^ for the case of the ACN molecule. The contour plot shows surface free charge density (*σ*_free_) at the experimental PZC of this system, *μ̃*_e_ = −4.98 eV,^33^ at varying *θ*_T_ and *ϵ*_op_^int^. Therefore, permissible values of *θ*_T_ and *ϵ*_op_^int^ are located at solid black dots corresponding to zero *σ*_free_.

Combining Kohn–Sham DFT simulations and a recent constant-potential method of modeling metal–solution interfaces,^15,30−32^ we investigate how aprotic solvents modulate the electron spillover at the metal–solution interface formed between Au(111) and KPF_6_ aprotic solutions, as shown in Figure 1a, how the solvent modulation varies at different electrode potentials, and the origin of the solvent dependency of the PZC observed in an experimental study.^33^ To examine the generality of the insights gained from our analysis of aprotic solvents, we extend our analysis to the case of water. The obtained understanding lays the basis for interpreting solvent effects on the activity of electrochemical reactions.

## Results

### Density-Potential Functional Approach of Modeling Electrochemical Interfaces: Theoretical Framework and First-Principles-Assisted Parametrization

The constant-potential approach combines an orbital-free DFT (OF-DFT) of metal electrons, a classical DFT of the electrolyte solution, and parametrized potential energy functions of metal–solution interactions.^15,30−32^ With the detailed derivation in ref (32), this method describes the metal–solution interface using two controlling equations in terms of the electron density *n*_e_ and the electrostatic potential *ϕ*
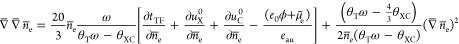
1
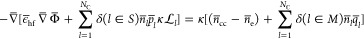
2where the overbar denotes variables and operators in the dimensionless system, ∇̃ = *a*_0_ ∇ with *a*_0_ (=Bohr radius) as the reference length, *n̅*_e_ = *n*_e_(*a*_0_)^3^,  with *e*_0_ as the elementary charge, *k*_B_ is the Boltzmann constant, and *T* is the temperature.  with *ϵ*_0_ as the permittivity of vacuum, , and *n̅*_cc_ are the dimensionless high-frequency (optical) permittivity, dipole moment of solvent, charge of electrolyte ions, and charge density of metal cationic cores, respectively. In eq 1, . *t*_TF_ is the kinetic energy functional, *u*_X_^0^ and *u*_C_^0^ are the exchange and correlation energy functionals of a homogeneous electron gas, respectively. *θ*_T_ and *θ*_XC_ are the gradient coefficients tuning the contribution of the “semilocal” term in kinetic energy and exchange–correlation energy, respectively. *μ̃*_e_ is the electrochemical potential of electrons. *e*_au_ = 27.2 eV is the energy in atomic units. In eq 2,  is a scalar number derived from fundamental constants, and  with *E̅* = |∇*ϕ̅*| being the dimensionless electric field. The symbol *δ*(*l* ∈ M) is equal to one for monopolar (M) charged particles, e.g., cations and anions, and zero otherwise, *δ*(*l* ∈ S) is equal to one for dipolar solvent molecules (S) and zero otherwise. Because the electron density and electrostatic potential are not dual variables as in the Kohn–Sham DFT but are two variables of equal status herein, we call this method a density-potential functional “theory” (DPFT).

The short-range interactions between the metal surface and solution particles are described using the Morse potential^34^
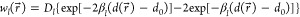
3with *D*_l_ being the well depth, *β*_l_, a coefficient controlling the well width, *d*(*r⃗*) the distance from *r⃗* to the metal surface, and *d*_0_ being the equilibrium distance between the molecule and the metal surface. When *r⃗* is within the metal, *d*(*r⃗*) is negative and *w*_l_(*r⃗*) becomes very positive, meaning that solution-phase particles have a negligible probability there. The parameters in *w*_l_ are determined from the Kohn–Sham DFT computations for the four aprotic solvents on the Au(111) surface, as shown in Figure S1. The binding energies are calculated in series of the distance between the solvent molecules and Au(111), while the *z*-coordinate of the solvent molecules is fixed. In Figures 1b and S2, these binding energy relations are fitted using Morse potentials with *R*^2^ values greater than 0.9. Because the Morse potential describes short-range electronic interactions and the interference of long-range electrostatic interactions should be avoided, we use the Ar atom that is iso-electronic to K^+^ for the case of K^+^ in Figure S3.^34^ As the PF_6_^–^ anion is a nonspecifically adsorbing species, its Morse potential is less important for the results, and we use the Morse potential of K^+^ for it. All the Morse potential parameters are compiled in Table S2, and it can be found that the distance between the Au(111) surface and solvent molecules decreases at larger *D*_1_’s, namely, stronger interactions.

The DPFT uses the Perdew–Burke–Ernzerhof (PBE)^35^ generalized gradient approximations for the exchange–correlation functional, as in the Kohn–Sham DFT calculations. Therefore, *θ*_T_ is the only free parameter in describing metal electrons. On the electrolyte solution side, the only free parameter is the interface high-frequency (optical) permittivity *ϵ*_op_^int^.^32^ We note that *θ*_T_ and *ϵ*_op_^int^ codetermine the electron spillover at the metal surface, and thus the PZC. The experimental PZCs of diglyme (DG), acetonitrile (ACN), dimethyl sulfoxide (DMSO), and propylene carbonate (PC) are 0.51, 0.44, 0.25, and 0.13 V (vs SHE), respectively.^33^ Provided these PZCs, *θ*_T_ and *ϵ*_op_^int^ cannot be varied independently, instead, they are correlated, as shown in Figures 1c and S4. In addition, *θ*_T_ should be universal for the Au(111) surface, while *ϵ*_op_^int^ is solvent specific.

So far, obtaining the optical permittivity *ϵ*_op_^int^ at metal–solution interfaces remains challenging. As a substitute, the bulk optical permittivity *ϵ*_op_^bulk^, which is equal to the square of the refractive index *n*, is widely used.^36^*n* can be calculated using the Lorentz–Lorenz equation^37^
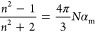
4here, *N* and *α*_m_ are the number density and polarizability of the solvent molecules, respectively. *α*_m_ can be determined by Kohn–Sham DFT calculations.^38^ The values of *α*_m_, *n*, and *ϵ*_op_^bulk^ are collected in Table S3. However, Hou et al.^39^ found that dielectric saturation occurs at metal–solution interfaces, resulting in an approximately constant *ϵ*_op_^int^ for all solvents examined in their work. This saturated permittivity determines the EDL capacitance. Herein, we use *θ*_T_ = 3.5 and determine the effective *ϵ*_op_^int^ values for DG, ACN, DMSO, and PC to be 5.49, 4.69, 4.70, and 5.07, respectively. These values closely align with the estimates reported by Hou et al.^39^

The key advantage of our DPFT model is its capability of simulating the metal–solution interface at constant electrochemical potential, tantamount to actually changing the electrochemical potential of electrons *μ̃*_e_ in eq 1
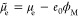
5with  being the chemical potential of a homogeneous electron gas. *ϕ*_M_ is the electric potential of the metal. Combining the ordinary differential equations (ODE) in eqs 1 and 2 result in them being solved with the following boundary conditions: ∇̅*n̅*_e_ = 0, ∇̅*ϕ̅* = 0 at the bulk metal; *n̅*_e_ = 0, *ϕ̅* = 0 at the bulk solution. Notably, we use the inner potential of the bulk electrolyte as the reference. Therefore, it is essential to incorporate the solvent’s surface potential into *μ̃*_e_. The surface potentials of the five solvents are collected in Table S4. The technical details of solving the model are provided in the section on computational methods.

### Constant-Potential, Spatially Resolved, Microscopic Structure of the Interface

**Figure 2:**
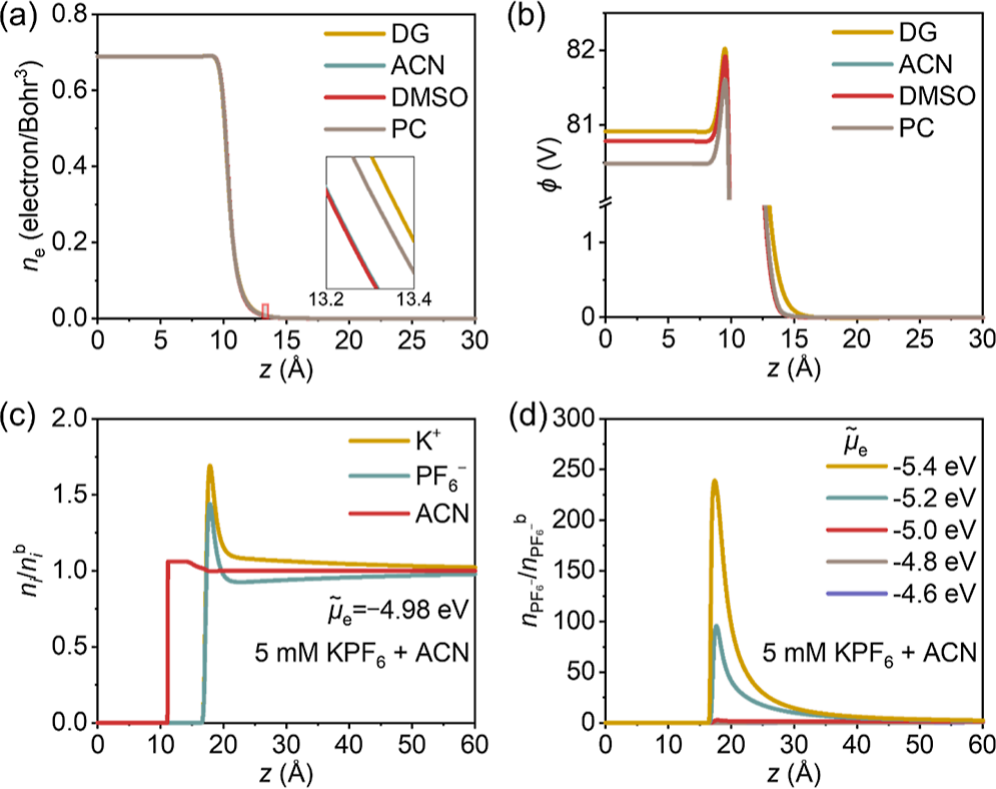
Constant-potential spatially resolved structures of the Au(111)–solution interface. The distribution of (a) electron density *n*_e_ and (b) electrostatic potential *ϕ* at the PZC in DG, ACN, DMSO, and PC. The inset of (a) is a close-up in the spillover region just outside the metal–solution interface. (c) Distribution of ions and solvent density at PZC (*μ̃*_e_ = −4.98 eV) in a solution of 5 mM KPF_6_ in ACN. (d) Dimensionless densities of PF_6_^–^ normalized to their bulk values as a series of electrochemical potential of electrons *μ̃*_e_ in solution of 5 mM KPF_6_ in ACN.

With above parametrization and setup of boundary conditions, the DPFT model enables us to solve for distributions of electron density *n*_e_, electrostatic potential ϕ, and ion concentrations at varying electrode potentials, as shown in Figure 2a–d. Such a capability lays the basis for studying how solvent modulates, *under constant potential conditions*, the electron spillover and changes the electrochemical properties of the metal–solution interface.

Figure 2a,b presents the distribution of *n*_e_ and *ϕ* in DG, ACN, DMSO, and PC at their respective PZCs. Interestingly, the electron spillover develops to different extents in these solvent molecules, thus, changing the potential distribution accordingly. Electrons spill out most in DG, and least in ACN, as seen in the inset of Figure 2a. Understanding the solvent-specific electron spillover is key to deciphering different PZCs of Au(111) in these solvent molecules, which will be analyzed in a later section.

Figure 2c displays the distribution of ions and solvent density at the PZCs in a solution of 5 mM KPF_6_ in ACN. Because the binding strength of solvent molecules on the metal surface is greater than that of solvated ions, solvent molecule approach closer to the metal surface than ions at the PZC. Figure 2d presents the dimensionless densities of PF_6_^–^ normalized to their bulk values at a series of *μ̃*_e_ in solution of 5 mM KPF_6_ in ACN. When the electrode potential shifts from below (*μ̃*_e_ > −4.98 eV) to above the PZC (*μ̃*_e_ < −4.98 eV), the metal surface gets positively charged, and the density of KPF_6_^–^ ions increases.

### Confirming the Aptness of the Model in Terms of Experimental Double Layer Capacitance Data

**Figure 3:**
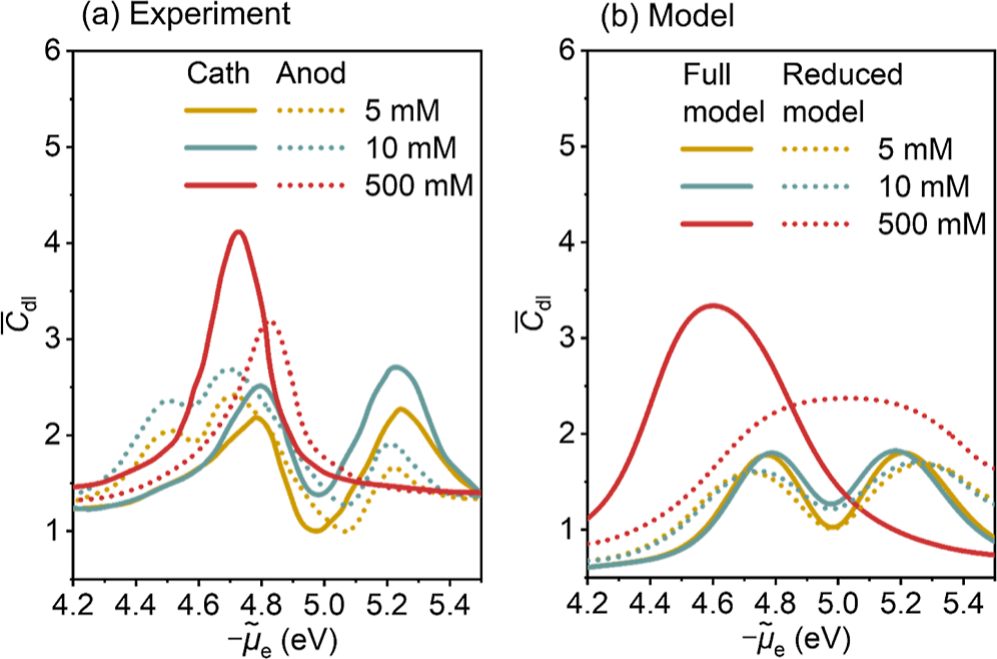
Differential double layer capacitance *C*_dl_ curves as a function of the electrochemical potential of electrons *μ̃*_e_. (a) Experimental *C*_dl_ of Au(111) electrode in *x* mM KPF_6_ (*x* = 5, 10, and 500) in ACN. Here, *C*_dl_ values are normalized to the capacitance at PZC at 5 mM KPF_6_ (17 μF/cm^2^). Experimental data were reported by Shatla et al.^33^ The solid and dashed lines represent cathodic and anodic scans, respectively. (b) Calculated *C*_dl_ of the full and reduced DPFT model of the Au(111) electrode in KPF_6_-ACN at different ion concentrations. Here, *C*_dl_ are normalized to the capacitance at PZC at the 5 mM KPF_6_ (8.99 μF/cm^2^). The reduced model neglects metal electrons and treats the metal surface as a boundary. The left and right peaks of the *C*_dl_ curves at low ion concentrations indicate crowding of solvated cations and solvated anions in the EDL, respectively.

By sweeping the model simulation in a range of electrode potentials, we calculate the *C*_dl_, an experimental measurable, from *σ*_free_.^32^ A comparison between the DPFT model and experiments in terms of *C*_dl_ profiles then leads credence to the DPFT model, forming the basis for later use of the DPFT model to decipher solvent-mediated electron spillover effects. Figure 3a,b shows the comparison between experimental and modeled *C*_dl_ at Au(111) electrodes at three concentrations (5, 10, and 500 mM) for Au(111) in a KPF_6_-ACN solution. To exclude the influence of metal surface roughness, *C*_dl_ is normalized to the capacitance at PZC at 5 mM KPF_6_. Experimental *C*_dl_ values calculated from both anodic and cathodic scans of cyclic voltammogram are shown in Figure 3a. Overall, the model results agree well with the experimental data, reproducing the camel-to-bell transition of *C*_dl_ profiles with increasing ion concentrations.^40,41^ The comparison of the other three aprotic solvents (DG, DMSO, and PC) is exhibited in Figure S5. The raw results of experiments and the model are also provided in Figure S6.

To illustrate the influence of metal electrons on *C*_dl_, we compare the full model and a reduced model that neglects metal electrons and treats the metal surface as an ideally metallic boundary. Two differences between the full model and the reduced model are noted. First, the full model gives a *C*_dl_ peak at 500 mM at a more negative electrode potential than the Gouy–Chapman minimums at 5 and 10 mM, while they coincide at the same PZC in the reduced model. This negative shift was observed experimentally in all types of solvent except PC.^33^ Considering the marked difference between experimental *C*_dl_ curves in anodic and cathodic scans, we shall not overinterpret this model-experiment discrepancy for PC. Second, *C*_dl_ is generally larger in the full model than in the reduced model. Both differences are ascribed to metal electronic effects.

Recently, the contribution of metal electronic effects to *C*_dl_ is described by the quantum capacitance or electronic capacitance *C*_q_, and it is connected in series with the capacitance of the electrolyte solution *C*_c_.^8,18,42^ It is important to note that in these models *C*_q_ accounts for change in the electronic structure of the bulk electrode, while the electron spillover effect is ignored. However, it is clear from our model that electron spillover creates an interfacial region where quantum mechanical electrons and classical ions are intertwined. Therefore, the prevailing serial model is problematic because it completely neglects the quantum–classical interaction region.

**Figure 4:**
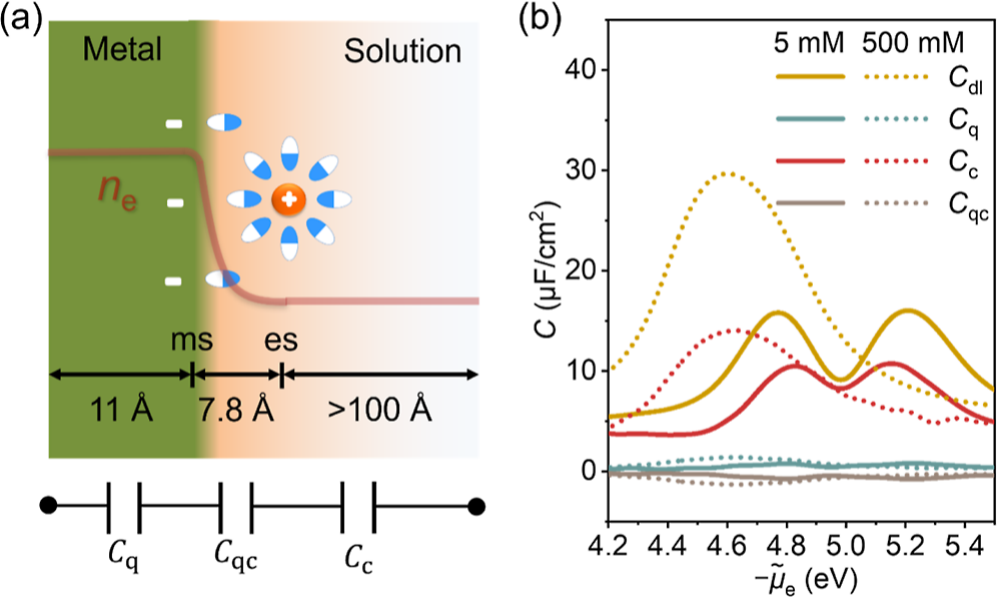
Differential capacitances of the metal–solution interface. (a) Schematic of the metal–solution interface. In the trichotomy model, we divide the total differential double layer capacitance *C*_dl_ into three constituent components: the quantum capacitance of the metal *C*_q_, the classical capacitance of the electrolyte solution *C*_c_, and the capacitance *C*_qc_ accounting for the interactions between metal electrons and electrolyte solution in proximity of the metal surface edge. (b) The calculated *C*_dl_, *C*_q_, *C*_qc_, and *C*_c_ for the Au(111) electrode in KPF_6_-ACN solution at 5 and 500 mM as a function of the electrochemical potential of electrons μ̃_e_. Here, *C*_q_ corresponds to the metallic region where the densities of ions and solvents is extremely low, namely,  in eq S8. *C*_c_ corresponds to the electrolyte solution region where the electron density is exceedingly small . *C*_qc_ corresponds to the middle region where both metal electrons and electrolyte species have a considerable density, which is calculated as .

As an improvement over the conventional dichotomy model, we propose a trichotomy model that divides *C*_dl_ into three components: *C*_q_, *C*_qc_, and *C*_c_ in series, as portrayed in Figure 4a. *C*_qc_ accounts for the interactions between metal electrons and electrolyte solution components in the proximity of the metal surface edge. Specifically, *C*_q_ corresponds to the metallic region where the densities of ions and solvent are extremely low, namely, . *C*_c_ corresponds to the electrolyte solution region where the electron density is exceedingly small . *C*_qc_ corresponds to the middle region, where both metal electrons and electrolyte species have a considerable density. Based on these density constraints, we can introduce two dividing planes: the metal surface edge (ms) and the plane beyond which  is met (es). Consequently, we decompose *C*_dl_ into
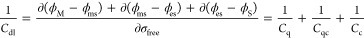
6where *ϕ*_ms_, *ϕ*_es_, and *ϕ*_S_ denote the electric potential at the ms, es, and bulk solution, respectively. According to eq 6, *C*_qc_ can be derived as . Using this trichotomy model, we calculate *C*_q_, *C*_c_, and *C*_qc_ at 5 and 500 mM in Figure 4b. Remarkably, it is observed that the value of *C*_qc_ is negative. This phenomenon stems from the fact that the potential drop (*ϕ*_ms_ – *ϕ*_es_) increases at more negative electrode potentials, corresponding to more negative *σ*_free_. This is because electron spillover is promoted at a more positive *μ̃*_e_.

### Insights into Solvent-Dependent PZC

Having demonstrated the importance of metal electronic effects and the efficacy of the DPFT model in describing them, we now address the key question posed at the beginning, viz., what is the main origin of solvent-dependent PZC?

**Figure 5:**
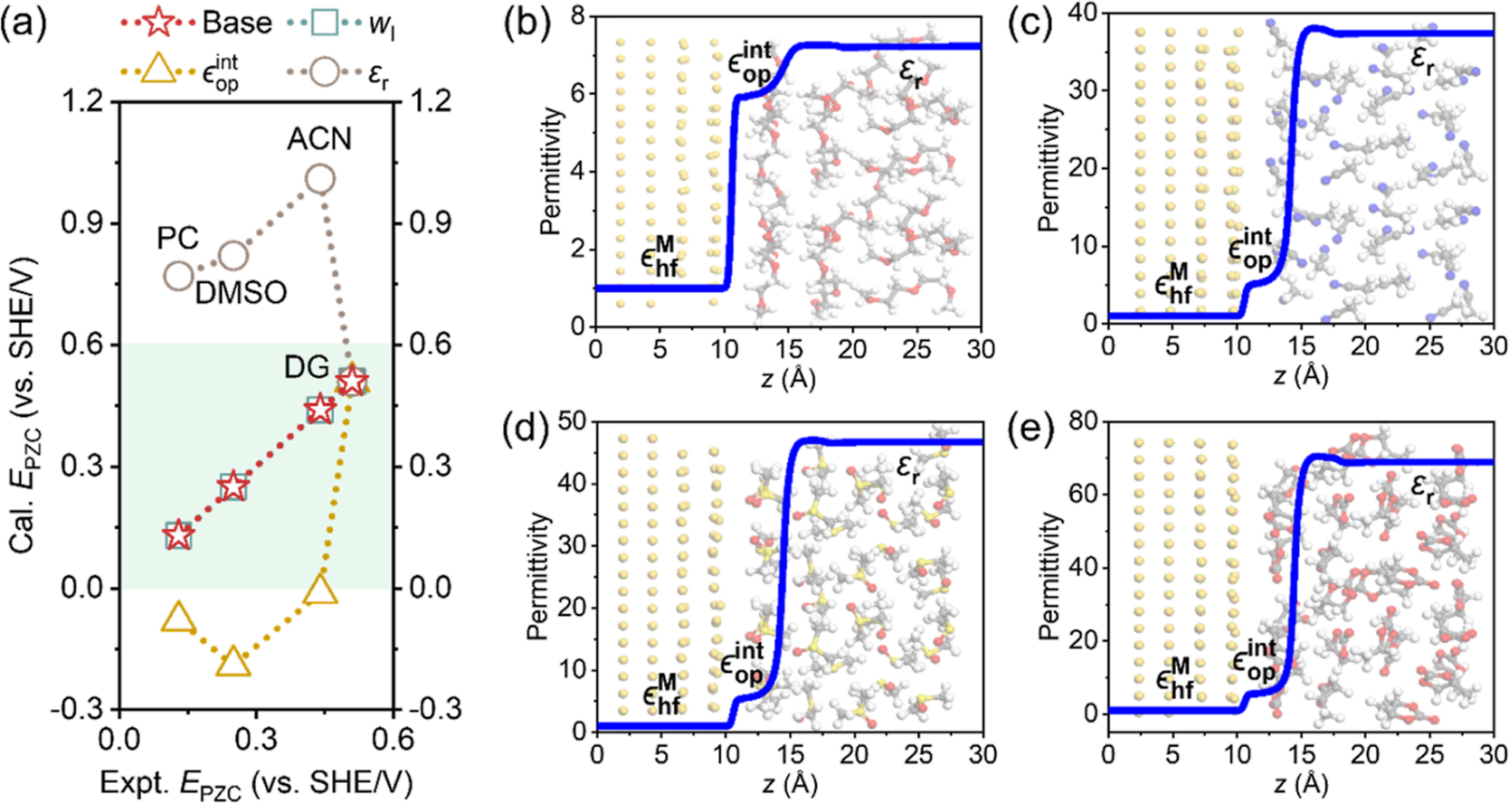
Solvent effects on PZC. (a) Effects of the optical permittivity *ϵ*_op_^int^, short-range metal–solvent interaction *w*_l_, and bulk permittivity ε_r_ of solvents on PZCs by comparing the calculated and experimental PZCs. The investigated solvents include DG, ACN, DMSO, and PC. The experimental PZC values were reported by Shatla et al.^33^ Here, we calculate the change in *E*_PZC_ due to a variation in these parameters using the one-variable-at-one-time method. For comparison, a base case, where four solvents maintain their original parameters, is included. The parametric analysis is conducted in the following manner. Considering the influence of *ϵ*_op_^int^. In a thought experiment, we use DG’s *ϵ*_op_^int^ for all solvents and calculate the PZCs, shown as triangles in Figure 5a. We then repeat the same procedure for *w*_l_ and *ε*_r_. The structure and total permittivity of the Au(111)–solution interface at the PZC are shown in (b) DG, (c) ACN, (d) DMSO, and (e) PC. *ϵ*_hf_^M^ is equal to one because all electrons are described explicitly and *ϵ*_hf_^M^ accounts for the polarizability of the nuclei. The snapshots of Au(111)–solvent molecular structures shown in the background are obtained from ab initio molecular dynamics simulations. The technical details of AIMD simulations are provided in the section on computational methods.

First, the influence of three key solvent properties in the DPFT model, including the optical permittivity *ϵ*_op_^int^, short-range metal–solvent interaction *w*_l_, and bulk permittivity *ε*_r_, on PZCs is analyzed. Specifically, we calculate the change in *E*_PZC_ due to a variation in these parameters using the one-variable-at-one-time method. For comparison, a base case, where four solvents maintain their original parameters, is included. Because model parameters for the base case are determined by fitting experimental PZCs, we find a 45° line in Figure 5a where the model-based PZCs are plotted against experimental PZCs. The parametric analysis is conducted in the following manner. Considering the influence of *ϵ*_op_^int^. In a thought experiment, we use DG’s *ϵ*_op_^int^ for all solvents and calculate the PZCs, shown as triangles in Figure 5a. We then repeat the same procedure for *w*_l_, and *ε*_r_.

Only a slight difference is observed when DG’s *w*_l_ is used for all solvents. On the contrary, marked differences are observed when DG’s *ε*_r_ and *ϵ*_op_^int^ are used invariantly for all solvents. Specifically, because DG’s *ε*_r_ is the smallest, see Figure 5b–e, increased PZCs of the other three solvents imply that the PZC is inversely related to *ε*_r_. In the same manner, because DG’s *ϵ*_op_^int^ is the largest, decreased PZCs of the other three solvents imply that the PZC is inversely related to *ϵ*_op_^int^. This parametric analysis reveals the importance of interfacial permittivity distribution in the electron spillover region on determining the PZC. This argument is further substantiated by the absence of any discernible relationship between the PZC and bulk solvent properties, such as *ε*_r_ and donor number, as shown in Figure S7.

To understand the effects of interfacial permittivity on the PZC, we shall first understand that on electron spillover. To quantify this dependency, we define *M*_e_^s^ as the moment of the electron density distribution
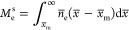
7with *x̅*_m_ denoting the metal boundary. A higher *M*_e_^s^ signifies a more extended distribution of the electron density. The *M*_e_^s^ values of DG, ACN, DMSO, and PC are calculated to be 0.522, 0.477, 0.476, and 0.508, respectively. DG with the largest *ϵ*_op_^int^ while the smallest ε_r_ has the largest *M*_e_^s^, namely, the most extended electron tail. The second largest *M*_e_^s^ is found at PC with the second largest *ϵ*_op_^int^ and the largest *ε*_r_. ACN (*ϵ*_op_^int^ = 4.69 and *ε*_r_ = 37.4) and DMSO (*ϵ*_op_^int^ = 4.70 and *ε*_r_ = 46.7) have similar *M*_e_^s^. Even though *ϵ*_op_^int^ varies within one among the four solvents, it has a decisive role in determining *M*_e_^s^ because the electron density decreases exponentially away from the metal surface, as seen in Figure 2a. The strong association between *M*_e_^s^ and *ϵ*_op_^int^ can be attributed to the dielectric screening effect of the repulsive interaction among electrons.^43,44^ When *ϵ*_op_^int^ is larger, the dielectric screening effect at the interface becomes more pronounced, suppressing electron repulsion and facilitating electron spillover.

It is interesting to note that DMSO has the narrowest electron tail but the second largest *ϵ*_op_^int^, together making its PZC the second largest. In addition, though the electron tail is more extended in PC than in ACN, the PZC is lower for the former because of the higher *ϵ*_op_^int^. In summary, *ϵ*_op_^int^ plays double-edged roles in determining the PZC. On the one hand, a higher *ϵ*_op_^int^ makes the electron tail more extended, tending to increase the PZC. On the other hand, a higher *ϵ*_op_^int^ decreases the surface potential of the metal surface, tending to decrease the PZC. Combined, the double-edged roles result in a nonmonotonic relationship between the PZC and *ϵ*_op_^int^ for the four solvent molecules.

### Applicability of the Obtained Understanding to Other Systems

**Figure 6:**
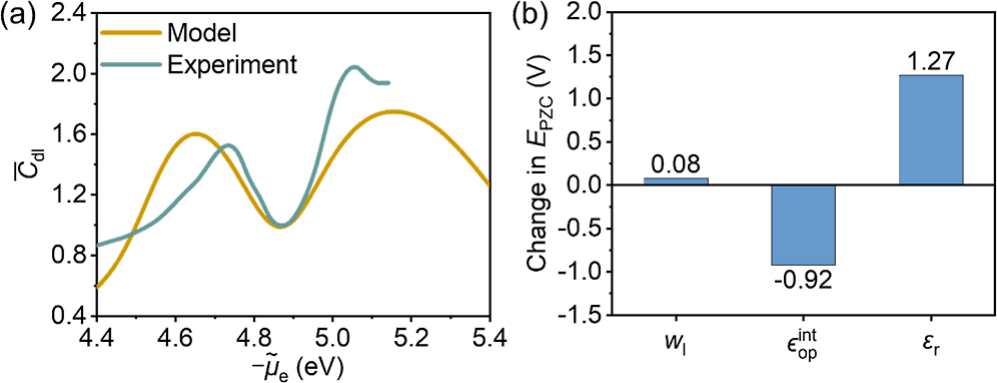
(a) Comparison between experimental and DPFT calculated differential capacitance of double layer *C*_dl_ at the Au(111) electrode in aqueous solution of KPF_6_ at a concentration of 10.87 mM. The *C*_dl_ curve is plotted as a function of the electrochemical potential of electrons *μ̃*_e_, which can be transformed into the electrode potential *ϕ*_M_ up to some constants. Experimental data were reported by Shatla et al.^33^*C*_dl_ are normalized to the capacitance at PZC. (b) Variations in the PZC of the Au(111)–aqueous solution interface when the short-range metal–solvent interaction *w*_l_, the optical permittivity *ϵ*_op_^int^, and the bulk permittivity *ε*_r_ are replaced with the counterparts of DG, respectively.

One might be curious about whether the understandings obtained at four aprotic solvents are applicable to water. To this end, we extend our investigation to the Au(111)–KPF_6_ aqueous interfaces. Following the same procedure of calibrating model parameters developed for the aprotic solvents, we determine an effective *ϵ*_op_^int^(=3.77) for the Au(111)–KPF_6_ aqueous interface at a concentration of 10.87 mM. The relatively low *ϵ*_op_^int^ observed at the Au(111)–aqueous solution interface could be attributed to the presence of hydrogen bonds.^45^ A comparison of the model-based and experimental *C*_dl_ profiles is illustrated in Figures 6 and S8. The fair agreement between the model and experiment supports the transferability of the DPFT model to aqueous solution systems. In addition to Au(111) studied in this work, Ag(111) has also been modeling using our DPFT approach in a previous study.^32^

Because water possesses the highest *ε*_r_ and the lowest *ϵ*_op_^int^, if we substitute water’s *ϵ*_op_^int^ with DG’s *ϵ*_op_^int^, we expect a negative shift in the PZC according to our understanding obtained at aprotic solvents. Similarly, if we substitute water’s *ε*_r_ with DG’s *ε*_r_, we expect a positive shift in the PZC. In accord with our analysis, Figure 6b shows a negative shift of −0.92 V from the substitution with DG’s *ϵ*_op_^int^ and a positive shift of 1.27 V from the substitution with DG’s *ε*_r_. In addition, a small positive shift of 0.08 V is obtained from the replacement of the short-range interactions. To conclude, the analysis on the Au(111)–aqueous solution interface further corroborates the pivotal role of the local permittivity in regulating electron spillover and PZC.

## Conclusions

In summary, we have studied the solvent dependency of the PZC at the Au(111)–KPF_6_ interfaces. To achieve this, we have employed a constant-potential DPFT model that was parametrized with first-principles calculations and experimental data. The origin of different PZCs of Au(111)–KPF_6_ in four different aprotic solvents has been analyzed in terms of solvent-modulated electron spillover. Double-edged effects of the local permittivity are revealed. Specifically, the electron spillover is facilitated through increased local permittivity, which arises from enhanced screening of electron–electron interactions and tends to increase the PZC. On the contrary, when the electron spillover is fixed, the PZC is lower at increased local permittivity due to enhanced screening of the space charge density. Therefore, the PZC exhibits a nonmonotonic relationship with the local permittivity. Our model captures the experimental *C*_dl_ curves at varying concentrations of KPF_6_. To comprehend why the capacitance peak in concentrated solutions is situated at a more negative potential than the Gouy–Chapman minimum in dilute solutions, it is crucial to consider the electronic effects of the metal. To gain insights into the contribution of electron spillover to the overall capacitance, we have decomposed the total capacitance into three components: the quantum capacitance of the metal *C*_q_, the classical capacitance of electrolyte solution *C*_c_, and the capacitance *C*_qc_ accounting for the interactions between metal electrons and electrolyte solutions in the proximity of the metal surface edge. Our findings reveal that *C*_qc_ assumes negative values, which can be attributed to the potential dependence of electron spillover. Shedding light on the origin and consequences of solvent-modulated electron spillover, the present work is instrumental to understanding solvent effects on the local reaction environment and electrocatalytic activities.

## Computational Methods

### Computational Details of Kohn–Sham DFT

We employed Kohn–Sham DFT calculations to optimize the geometries of the solvent molecules on Au(111) and calculated their binding energies. All calculations were performed using the Vienna ab initio simulation package (VASP).^46,47^ The PBE exchange–correlation functional together with the projected augmented wave potentials was used.^48,49^ The D3 approach within Grimme’s formalism was used for nonlocal van der Waals interactions.^50,51^ A plane-wave basis set with a cutoff energy of 500 eV was used to expand the eigenstates of the electron wave functions. The Au(111) surface was constructed by using the periodic slabs consisting of five atomic layers. A vacuum layer of 20 Å in the vertical direction to the model slab was set to avoid the lateral interactions between the layer and its images. To maximize computational efficiency while not affecting the calculation accuracy, a minimal Gamma 2 × 2 × 1 *k*-point grid was used to sample the Brillouin zones for all slab calculations. The structures were optimized until the total energies and atomic forces were converged to within 0.001 meV and 0.02 eV/Å, respectively.

### Computational Details of DPFT

All DPFT simulations were solved by using COMSOL Multiphysics. The two controlling equations, eqs 1 and 2, were implemented as a coefficient-form ODE. All parameters used in the DPFT simulations are summarized in Tables S1 and S2. In this work, we used the following initial guess for the electron density and its gradient
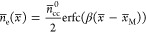
8
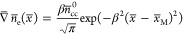
9where  with *N*_Au_ = 79 representing the number of electrons of a gold atom, and *a*_Au_ = 4.08 Å is the lattice constant of the cubic closed-packed cell of Au, which contains four gold atoms, and *a*_0_ = 0.529 Å is the Bohr radius. Correspondingly, the initial guess for the electric potential was obtained from solving the Poisson equation –∇̅^2^*ϕ̅* = κ(*n̅*_cc_ – *n̅*_e_)
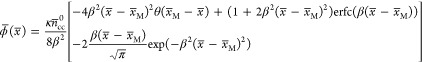
10and the corresponding potential gradient reads
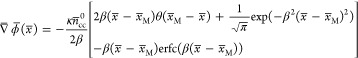
11

Here, *θ*(*x̅*_M_ – *x̅*) is a Heaviside function, which is equal to 1 when *x̅* < *x̅*_M_ and zero elsewise.

### Parameterization of DPFT Model: *θ*_T_, *ϵ*_op_^int^

The high-frequency (optical) permittivity *ϵ*_hf_ usually varies spatially because the electrode and electrolyte have different optical permittivities, denoted as *ϵ*_hf_^M^ and *ϵ*_op_^int^, respectively. *ϵ*_hf_ is interpolated as
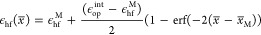
12

In this work, the DPFT model considers all-electron calculation, so *ϵ*_hf_^M^ = 1 is used. Because the orientational polarization of solvent is considered, *ϵ*_op_^int^ is determined by the electronic polarization of ions and solvent molecules at the interface. *ϵ*_op_^int^ is the only free parameter for the electrolyte solution. *θ*_T_ is the only free parameter for describing metal electrons. Therefore, we fix the electrochemical potential of electrons according to the experimental PZC values and then sweep *θ*_T_ and *ϵ*_op_^int^ simultaneously. The contour plots in Figure S4 show surface charge density *σ*_free_ at the experimental PZC of these systems at varying *θ*_T_ and *ϵ*_op_^int^. Therefore, permissible values of *θ*_T_ and *ϵ*_op_^int^ are located at solid black dots corresponding to zero *σ*_free_. These black points can be fitted with straight lines.

### Computational Details of Differential Double Layer Capacitance *C*_dl_

The differential double layer capacitance, i.e., potential-dependent capacitance, can be calculated as
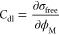
13where *σ*_free_ is the excess free charge density and *ϕ*_M_ is the electric potential of the bulk metal. The *σ*_free_ of the EDL is defined as
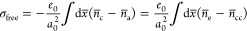
14the second equal here sign is because the whole EDL is electroneutral, and the factor  is the result of dimensional balance. The substitution of eqs 14 into 13 gives the following equation for calculating the *C*_dl_
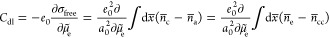
15

### Computational Details of ab initio Molecular Dynamics

The ab initio molecular dynamics simulations (AIMD) were implemented in CP2K/Quickstep.^52^ The Goedecker–Teter–Hutter (GTH) pseudopotentials were employed to describe all atoms.^53,54^ The DZVP-MOLOPT-SR-GTH Gaussian basis set was applied to all types of atoms.^55^ To ensure accuracy, the plane wave energy cutoff was set to 350 Ry. The exchange–correlation effect was described by the PBE functional.^35^ The AIMD simulations were carried out in the canonical ensemble (*NVT*), maintaining a target temperature of 300 K with a time step of 0.5 fs. To speed up the simulations, the second-generation Car–Parrinello MD (SGCP MD)^56^ was employed. In the corrector step of SGCP MD, the maximum number of iterations in the self-consistent field loop was set to 5. The Langevin friction coefficient γ_D_ was set to 0.0001 fs^–1^, while the intrinsic friction coefficient γ_L_ was set to 2.2 × 10^–4^ fs^–1^ for solvents and 5 × 10^–5^ fs^–1^ for Au.

## Data Availability

The data that support the findings of this study are available from the corresponding author upon reasonable request.

## Supporting Information Available

Parameterization of the Morse potential; comparison between experimental and DPFT-calculated differential double layer capacitance; relationships of the potentials of zero charge for Au(111)–solution interface with solvent properties; and DPFT model parameters; surface potentials of solvent (PDF)
