# Supplementary material for: Origin of Solvent Dependency of the Potential of Zero Charge
Source: JACS Au. 2023 Nov 15;3(12):3381–90. doi: 10.1021/jacsau.3c00552 (PMC10751779; doi:10.1021/jacsau.3c00552)
Supplement: Supplementary file 1 — au3c00552_si_001.pdf [file au3c00552_si_001.pdf]

Supporting information for

Origin of Solvent Dependency of the Potential of Zero Charge

Weiqiang Tang[1, 2], Shuangliang Zhao[1, 3, *], and Jun Huang[2, 4, *]

[1] *State Key Laboratory of Chemical Engineering and School of Chemical Engineering, East China University of Science and Technology, Shanghai, 200237, China*

[2] *Institute of Energy and Climate Research, IEK-13: Theory and Computation of Energy Materials, Forschungszentrum Jülich GmbH, 52425 Jülich, Germany*

[3] *Guangxi Key Laboratory of Petrochemical Resource Processing and Process Intensification Technology and School of Chemistry and Chemical Engineering, Guangxi University, Nanning, 530004, China*

[4] *Theory of Electrocatalytic Interfaces, Faculty of Georesources and Materials Engineering, RWTH Aachen University, 52062 Aachen, Germany*

* To whom correspondence and requests for materials should be addressed. (Email: szhao@ecust.edu.cn, ju.huang@fz-juelich.de)

This PDF file includes:

Supplementary Figures S1 to S8

Supplementary Tables S1 to S4

Supplementary References

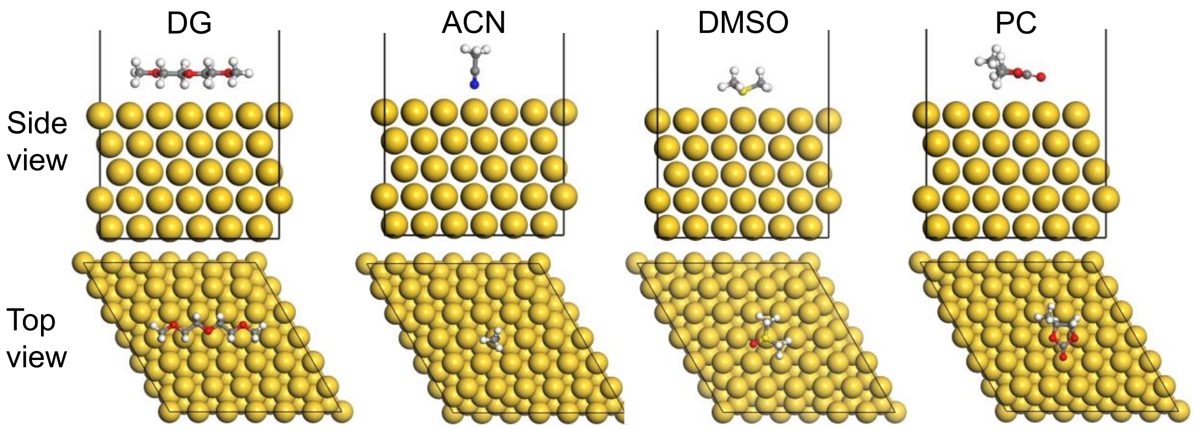

Supplementary Fig. S1. The optimal solvent conformations of diglyme (DG), and acetonitrile (ACN), dimethyl sulfoxide (DMSO), propylene carbonate (PC) on Au(111) surface. The deep yellow, white, grey, red, blue, and yellow balls represent Au, H, C, O, N, and S atoms, respectively.

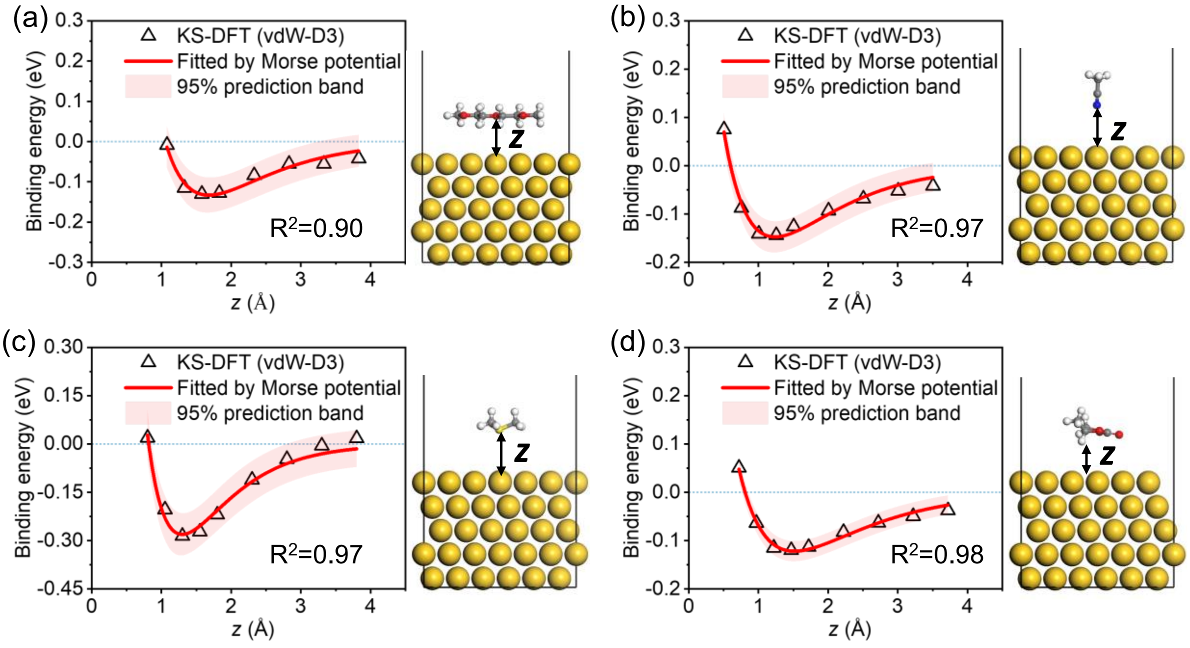

Supplementary Fig. S2. Parameterization of the Morse potential between Au(111) and (a) diglyme (DG), (b) acetonitrile (ACN), (c) dimethyl sulfoxide (DMSO), and (d) propylene carbonate (PC). The empty triangles indicate the calculated binding energy from Kohn-Sham DFT in each case, while the solid red lines are the fitted Morse potentials. The deep yellow, white, grey, red, blue, and yellow balls represent Au, H, C, O, N, and S atoms, respectively.

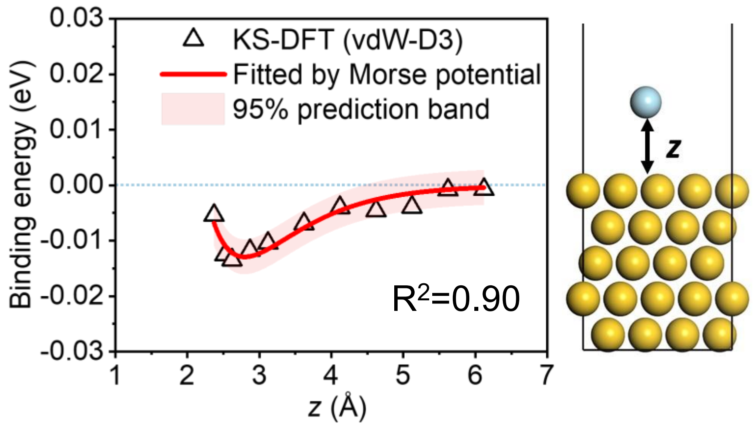

Supplementary Fig. S3. Parameterization of the Morse potential between Au(111) and Ar atom. The empty triangles indicate the calculated binding energy from Kohn-Sham DFT, while the solid red line is the fitted Morse potentials.

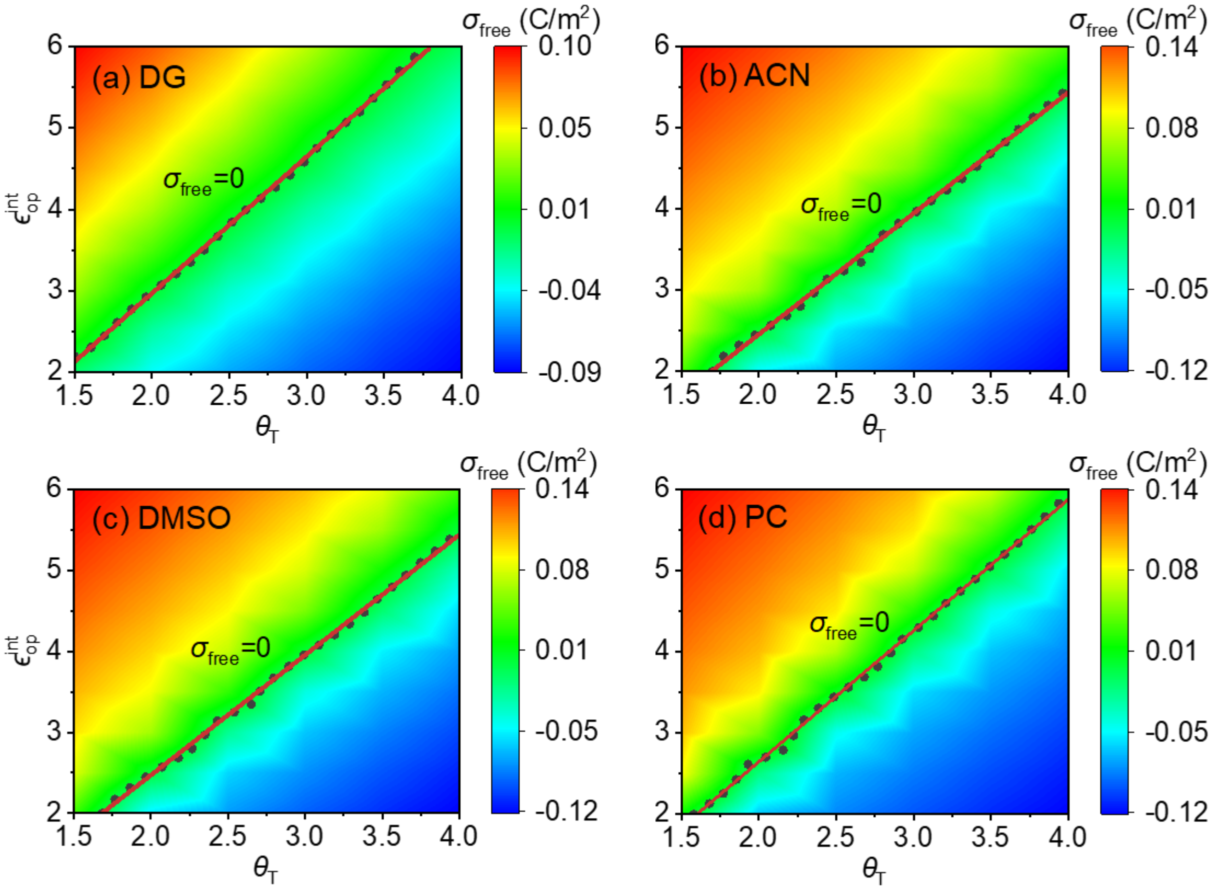

Supplementary Fig. S4. Determination of the θ_{T} and $\epsilon_{\mathrm{op}}^{\mathrm{int}}$ for the case of (a) diglyme (DG), (b) acetonitrile (ACN), (c) dimethyl sulfoxide (DMSO), and (d) propylene carbonate (PC) molecule. The contour plot shows surface charge density (σ_{free}) at the experimental PZC of these systems at varying θ_{T} and $\epsilon_{\mathrm{op}}^{\mathrm{int}}$. Therefore, permissible values of θ_{T} and $\epsilon_{\mathrm{op}}^{\mathrm{int}}$ are located at solid black dots corresponding to zero σ_{free}. The solid red lines are the fitted lines, and the equations and values of R^2 are shown below each plot.

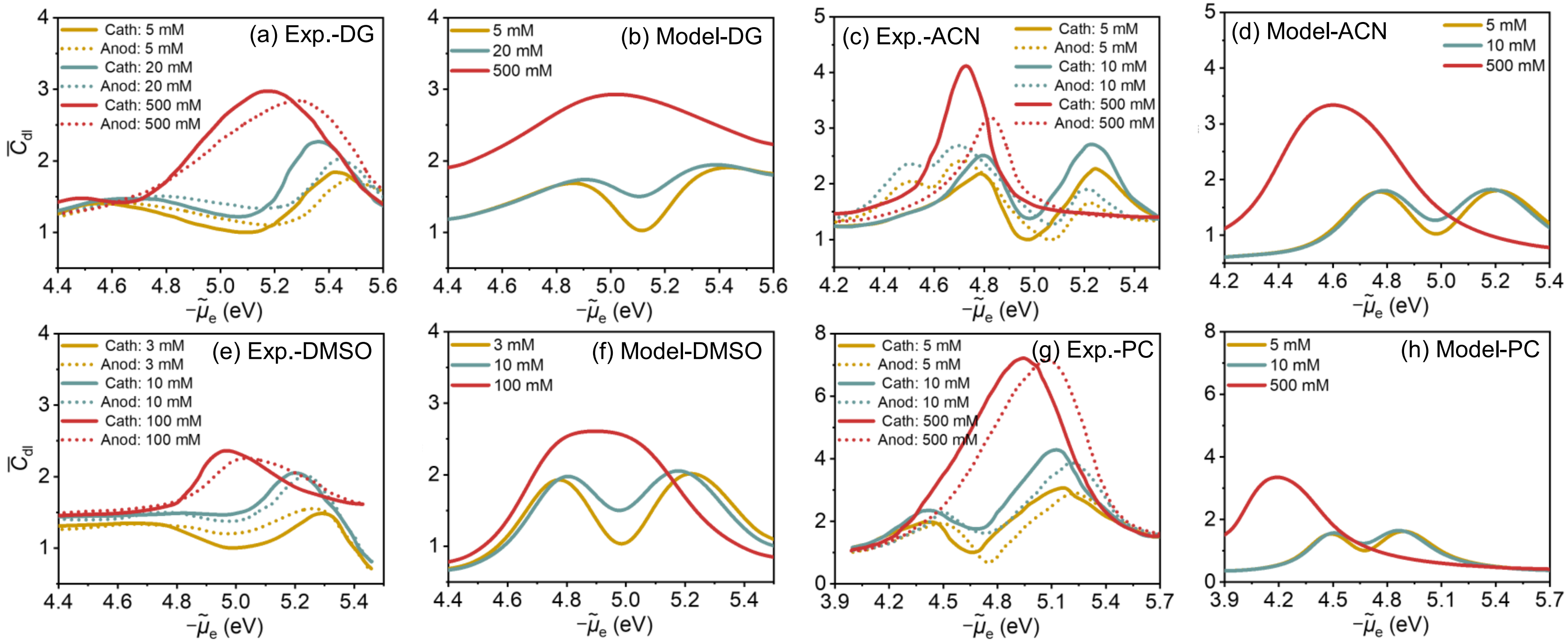

Supplementary Fig. S5. Comparison between experimental and DPFT calculated differential double layer capacitance C_{dl} at Au(111) electrode in different concentrations solutions of KPF_6 in diglyme (DG), acetonitrile (ACN), dimethyl sulfoxide (DMSO), and propylene carbonate (PC). C_{dl} curve is a function of the electrochemical potential of electrons $\tilde{\mu}_{\mathrm{e}}$, which can be transformed to the electrode potential ϕ_{M} up to some constants. C_{dl} are normalized to the capacitance at PZC using the minimum KPF_6 concentration of 5mM for DG, ACN, and PC, and 3mM for DMSO. Experimental data were reported by Shatla *et al*.[1] In the experimental data, the solid and dashed lines represent cathodic and anodic scans, respectively.

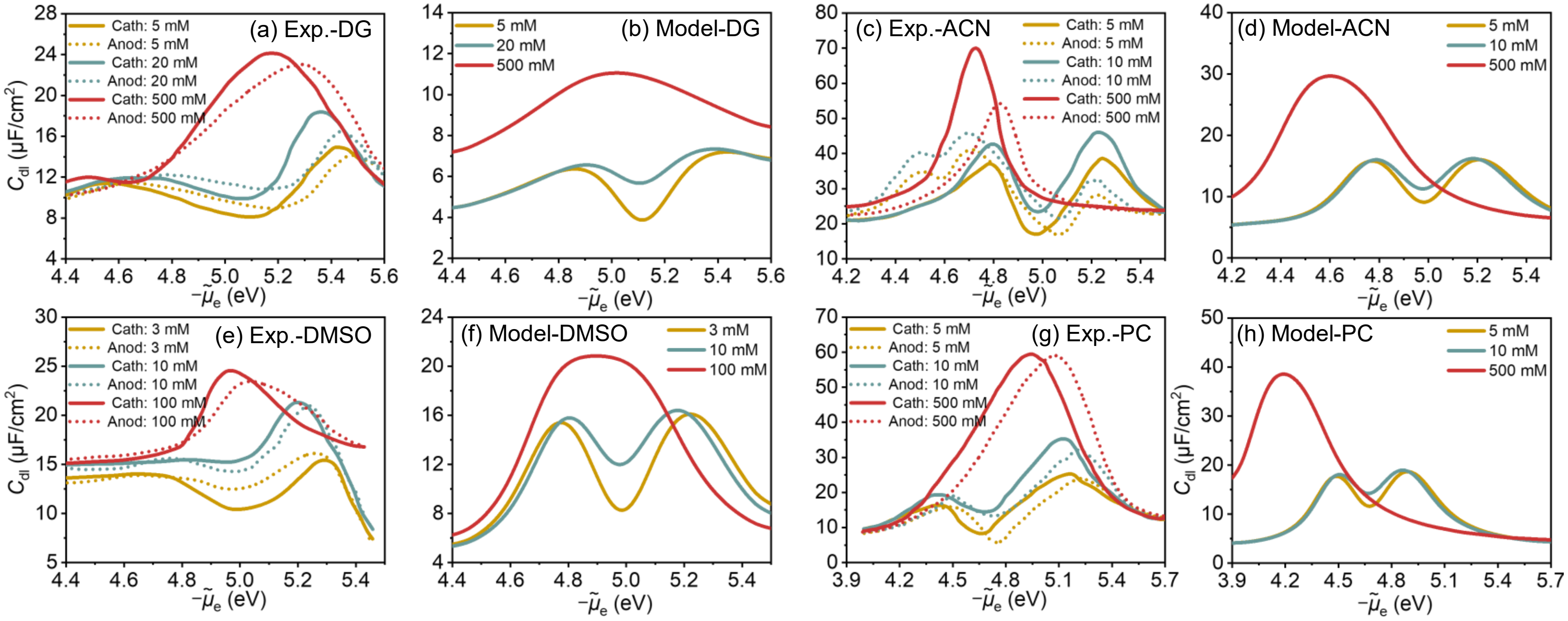

Supplementary Fig. S6. Comparison between experimental and DPFT calculated differential double layer capacitance $C_{\rm dl}$ at Au(111) electrode in different concentrations solutions of KPF_6 in diglyme (DG), acetonitrile (ACN), dimethyl sulfoxide (DMSO), and propylene carbonate (PC). $C_{\rm dl}$ curve is a function of the electrochemical potential of electrons $\tilde{\mu}_{\rm e}$, which can be transformed to the electrode potential $\phi_{\rm M}$ up to some constants. Experimental data were reported by Shatla *et al*.[1] In the experimental data, the solid and dashed lines represent cathodic and anodic scans, respectively.

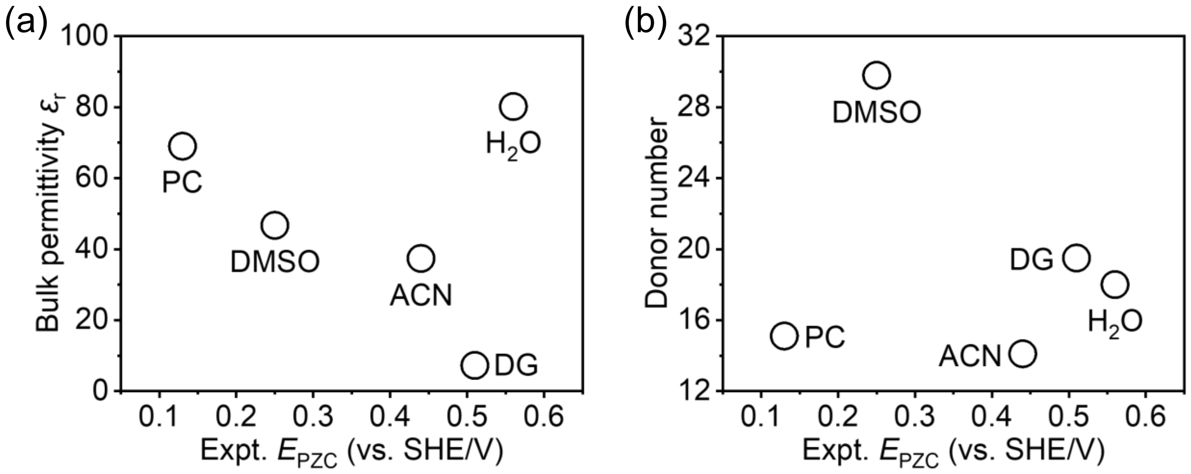

Supplementary Fig. S7. Relationships of the potentials of zero charge for Au(111)-solution interface with solvent properties, including (a) bulk permittivity ε_r and (b) donor number of the diglyme (DG), acetonitrile (ACN), dimethyl sulfoxide (DMSO), and propylene carbonate (PC). The experimental PZC values were reported by Shatla *et al*.[1]

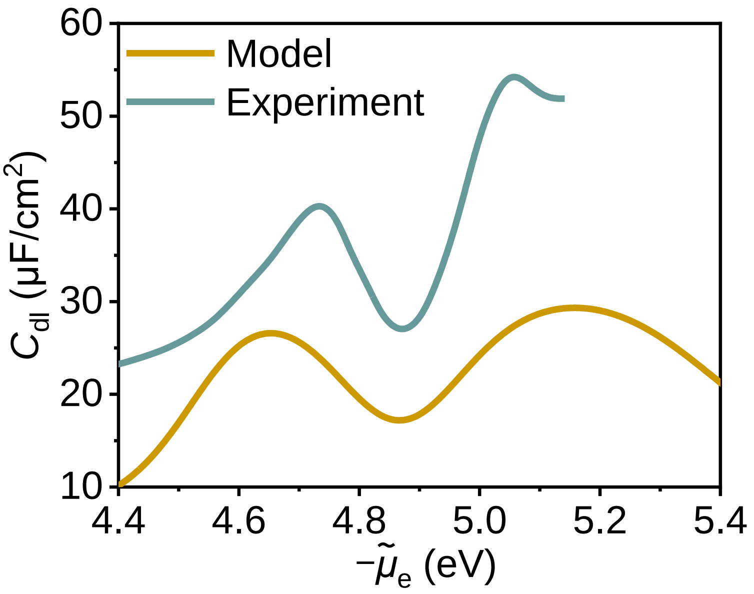

Supplementary Fig. S8. Comparison between experimental and DPFT-calculated differential capacitance of double layer C_{dl} at Au(111) electrode in aqueous solution of KPF_6 at a concentration of 10.87 mM. C_{dl} curve is plotted as a function of the electrochemical potential of electrons $\tilde{\mu}_{\mathrm{e}}$, which can be transformed to the electrode potential ϕ_{M} up to some constants. Experimental data were reported by Shatla *et al.*[1]

Supplementary Table S1. DPFT model parameters

Category	Symbol	Item	Value
General constants	R	Ideal gas constant	$8.314\ JK^{-1}mol^{-1}$
	k_B	Boltzmann constant	$1.38 \cdot 10^{-23}\frac{J}{K}$
	T	Temperature	298.15 K
	e0	Elementary charge	$1.6 \times 10^{-19}C$
	eau	Energy constant from arb. units to SI	27.2e0
	N_A	Avogadro's number	$6.02 \times \frac{10^{23}}{mol}$
	ϵ_0	Vacuum permittivity	$8.85 \times \frac{10^{-11}F}{m}$
	a_0	Bohr radius	$5.29 \times 10^{-11}\ m$
	n_{ref}	Reference number density	$(a_0)^{-3}$
	κ	Dimensionless constant	$\frac{e_0^2}{k_B T \epsilon_0 a_0}$
	z_a	Charge number of anions	-1
	z_c	Charge number of cations	1
	$\bar{n}_s^{DG}$	DG number density	$6.9 \times 10^3 N_A(a_0)^3$
	$\bar{n}_s^{ACN}$	ACN number density	$1.91 \times 10^4 N_A(a_0)^3$
	$\bar{n}_s^{DMSO}$	DMSO number density	$1.41 \times 10^4 N_A(a_0)^3$
	$\bar{n}_s^{PC}$	PC number density	$1.18 \times 10^4 N_A(a_0)^3$
	$\bar{n}_s^{H_2O}$	H_2O number density	$5.54 \times 10^4 N_A(a_0)^3$
	d_s	Diameter of solvent	$1 \times 10^{10} a_0 \left(\frac{1}{\bar{n}_s}\right)^{\frac{1}{3}}$ Å
	r_c	Radius of solvated cation	$(1.38 + d_s)$ Å
	r_a	Radius of solvated anion	$\left(3 + \frac{d_s}{2}\right)$ Å
	γ_c	Relative size of solvated cations	$(2r_c/d_s)^3$
	γ_a	Relative size of solvated anions	$(2r_a/d_s)^3$
	ε_r^{DG}	Bulk permittivity of DG	7.23
	ε_r^{ACN}	Bulk permittivity of ACN	37.4
	ε_r^{DMSO}	Bulk permittivity of DMSO	46.8

	ε_r^{PC}	Bulk permittivity of PC	69
	$\varepsilon_r^{H_2O}$	Bulk permittivity of H_2O	80.2
	p_s	Solvent dipole moment	$\left[\frac{3 * \left(\varepsilon_r - \epsilon_{op}^{int}\right)\epsilon_0 k_B T}{n_s N_A}\right]$ D
Metal	$\bar{n}_{cc}^0$	Dimensionless metal electron density	0.689
	$\bar{\epsilon}_{op}^M$	Optical dielectric constant of metal	1

Supplementary Table S2. Morse potential parameters for Au(111)-solution interactions.

System	D_l (eV)	β_l (Å^{-1})	d_0 (Å)
Au(111)–DG	0.13291	1.11362	2.54872
Au(111)–ACN	0.14732	1.0857	2.36022
Au(111)–DMSO	0.28047	1.44181	2.02349
Au(111)–PC	0.12184	0.98097	2.56091
Au(111)–H_2O	0.11975	1.13033	1.49630
Au(111)–Ar	0.01293	1.22895	2.79571

Supplementary Table S3. The calculated polarizability α_{m}, refractive index n, bulk optical permittivity $\epsilon_{\mathrm{op}}^{\mathrm{bulk}}$, and saturated permittivity $(\varepsilon_{\mathrm{r}})_{\mathrm{sat}}$ [2] of diglyme (DG), acetonitrile (ACN), dimethyl sulfoxide (DMSO), and propylene carbonate (PC).

Solvent	α_{m} (Å^3)	n^a	$\epsilon_{\mathrm{op}}^{\mathrm{s}}$	$(\varepsilon_{\mathrm{r}})_{\mathrm{sat}}$
DG	14.01	1.40 (1.41)	1.97	/
ACN	4.37	1.34 (1.34)	1.80	5.0
DMSO	7.93	1.48 (1.48)	2.18	5.7
PC	8.42	1.41 (1.42)	2.00	6.4

[a] The values in parentheses are from https://www.chembk.com/en.

Supplementary Table S4. The surface potential of water (H_2O), diglyme (DG), acetonitrile (ACN), dimethyl sulfoxide (DMSO), and propylene carbonate (PC).[3]

Solvent	χ (V)	Source
H_2O	0.13	Ref. 15
DG	-0.16	Estimated based on donor number
ACN	-0.10	Ref. 15
DMSO	-0.29	Ref. 15
PC	-0.11	Estimated based on donor number

Supplementary References

1. Shatla, A. S.; Landstorfer, M.; Baltruschat, H., On the differential capacitance and potential of zero charge of Au (111) in some aprotic solvents. *ChemElectroChem* **2021,** 8, (10), 1817-1835.
2. Hou, Y.; Aoki, K. J.; Chen, J.; Nishiumi, T., Solvent Variables Controlling Electric Double Layer Capacitance at the Metal–Solution Interface. *The Journal of Physical Chemistry C* **2014,** 118, (19), 10153-10158.
3. Trasatti, S., Interfacial behaviour of non-aqueous solvents. *Electrochimica Acta* **1987,** 32, (6), 843-850.
